# Trackins (Trk-Targeting Drugs): A Novel Therapy for Different Diseases

**DOI:** 10.3390/ph17070961

**Published:** 2024-07-19

**Authors:** George N. Chaldakov, Luigi Aloe, Stanislav G. Yanev, Marco Fiore, Anton B. Tonchev, Manlio Vinciguerra, Nikolai T. Evtimov, Peter Ghenev, Krikor Dikranian

**Affiliations:** 1 Departments of Anatomy and Cell Biology and Translational Stem Cell Biology, Research Institute, Medical University, 9002 Varna, Bulgaria; 2 Fondazione Iret, Tecnopolo R. Levi-Montalcini, Ozzano dell’Emilia, 40064 Bologna, Italy; 3 Institute of Neurobiology, Bulgarian Academy of Sciences, 1113 Sofia, Bulgaria; 4 Institute of Biochemistry and Cell Biology, National Research Council, IBBC-CNR, 00185 Rome, Italy; 5 Department of Translational Stem Cell Biology, Research Institute, Medical University, 9002 Varna, Bulgaria; 6 Department of Urology, University St Anna Hospital, 9002 Varna, Bulgaria; 7 Department of General and Clinical Pathology, Medical University, 9002 Varna, Bulgaria; 8 Department of Neuroscience, Medical School, Washington University, St. Louis, MO 63110, USA

**Keywords:** Trk-targeting drugs (trackins), Trk receptors, NGF, proNGF, BDNF, NT-3, cardiometabolic diseases, Alzheimer’s disease, cancer, pain

## Abstract

Many routes may lead to the transition from a healthy to a diseased phenotype. However, there are not so many routes to travel in the opposite direction; that is, therapy for different diseases. The following pressing question thus remains: what are the pathogenic routes and how can be they counteracted for therapeutic purposes? Human cells contain >500 protein kinases and nearly 200 protein phosphatases, acting on thousands of proteins, including cell growth factors. We herein discuss neurotrophins with pathogenic or metabotrophic abilities, particularly brain-derived neurotrophic factor (BDNF), nerve growth factor (NGF), pro-NGF, neurotrophin-3 (NT-3), and their receptor Trk (tyrosine receptor kinase; pronounced “track”). Indeed, we introduced the word *trackins*, standing for Trk-targeting drugs, that play an agonistic or antagonistic role in the function of TrkB^BDNF^, TrkC^NT−3^, TrkA^NGF^, and TrkA^pro-NGF^ receptors. Based on our own published results, supported by those of other authors, we aim to update and enlarge our *trackins concept*, focusing on (1) agonistic trackins as possible drugs for (1a) neurotrophin-deficiency cardiometabolic disorders (hypertension, atherosclerosis, type 2 diabetes mellitus, metabolic syndrome, obesity, diabetic erectile dysfunction and atrial fibrillation) and (1b) neurodegenerative diseases (Alzheimer’s disease, Parkinson’s disease, and multiple sclerosis), and (2) antagonistic trackins, particularly TrkA^NGF^ inhibitors for prostate and breast cancer, pain, and arrhythmogenic right-ventricular dysplasia. Altogether, the druggability of TrkA^NGF^, TrkA^pro-NGF^, TrkB^BDNF^, and TrkC^NT−3^ receptors via trackins requires a further translational pursuit. This could provide rewards for our patients.

## 1. Introduction

*Thus, the task is not so much to see what no one has yet seen but to think what nobody has yet thought about that which everybody sees.*

Arthur Schopenhauer

The discovery of nerve growth factor (NGF) in 1951 by Rita Levi-Montalcini was the Rosetta stone in understanding neural differentiation, survival, and functions [1,2]. Life, at both the local and systemic levels, requires nutritional, immune, neurotrophic, and metabotrophic support. Many routes may lead to the transition from a healthy to a diseased phenotype. However, there are not so many routes to travel in the opposite direction; that is, therapies for cardiometabolic diseases (CMD), neurodegenerative diseases, and cancers, thus extending human life expectancy and Quality of Life (QoL) [3,4,5,6]. The following pressing question thus remains: what are the pathogenic routes and how can they be counteracted for therapeutic purposes?

## 2. Neurotrophins and Their Receptors

At present, the neurotrophin family of proteins consists of NGF, pro-NGF, brain-derived neurotrophic factor (BDNF), pro-BDNF, neurotrophin-3 (NT-3), NT-4/5, and NT-6 [5,6]. Of these, NGF, pro-NGF, BDNF, and NT-3 are multifunctional proteins, which, in addition to their neurotrophic action, exert various extraneuronal effects directed to immune, endothelial, beta-pancreatic, muscle, epithelial, and other nonneuronal cells [3,4,5,6,7,8,9,10]. As well as the metabolism of lipids and carbohydrates, we named metabotrophic effects and metabotrophic factors (MTF) [5,6].

**Figure 1:**
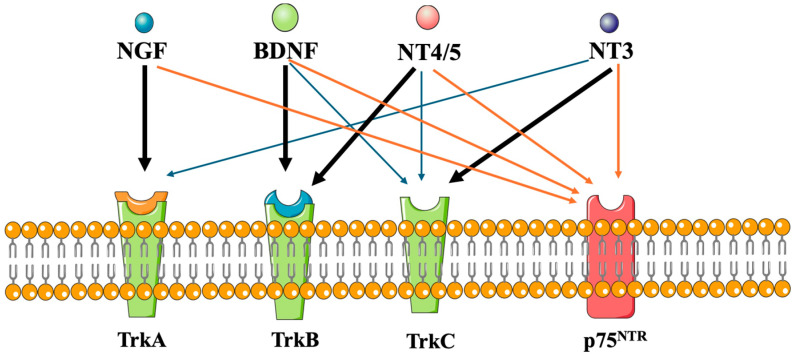
Neurotrophins and their Trk receptors. Redrawn from [11].

Neurotrophins elicit their outcomes via ligation to p75^NTR^), the pan-neurotrophin receptor, and Trk receptors, namely, TrkA^NGF^, TrkA^pro-NGF^, TrkB^BDNF^, TrkB^NT−4/5^, and TrkC^NT−3^. The acronym Trk intends for tyrosine receptor kinases vs. non-receptor tyrosine kinases, which have no transmembrane domain (Figure 1, Table 1, Table 2 and Table 3).

**Figure 2:**
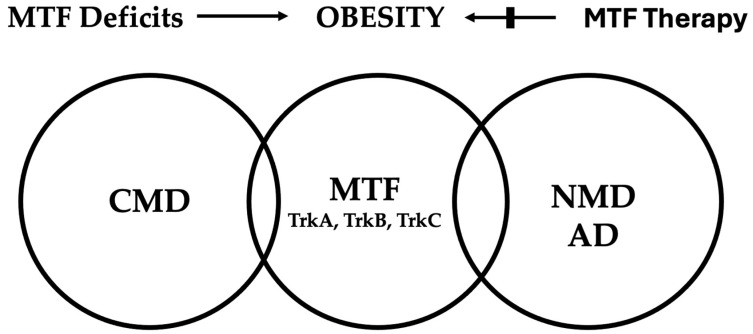
Metabotrophic factors (MTF) and their Trk receptors on the crossroads of the pathogenesis of and therapy for cardiometabolic diseases (CMD) and neurometabolic diseases (NMD), particularly Alzheimer’s disease (AD). Credit Nikifor N. Chaldakov.

In this connection, Figure 2 illustrates our own results of the potential significance of reduced local and/or blood levels of NGF and BDNF, functioning as metabotrophic factors (MTF) for the pathobiology of obesity and its related cardiometabolic and neurodegenerative diseases, particularly Alzheimer’s disease (AD), with the latter being considered a neurometabolic disease [3,4,5,6,8,9,10,18].

## 3. NGF, BDNF, and Their Trk Receptors: Druggable Targets for Disease Therapies

Druggability is a term used in drug discovery to describe biological targets [71,72]. In the context of the present article, these are the neurotrophins and their Trk receptors that are known or predicted to bind with high affinity to a drug [71,72]. Furthermore, by definition, the binding of the drug to a druggable target must alter the function of the target, with a therapeutic benefit to the patient [71,72]. The idea of druggability is most often constrained to low-molecular-weight chemicals (pharmaceuticals) but has also been revised to include biologicals such as therapeutic monoclonal antibodies, and nutraceuticals such as polyphenols extracted from vegetables [73,74].

There are numerous pathways that can cause the transition from a healthy to a diseased phenotype. In contrast, the pathways to reverse this process, such as treating conditions like CMD and cancers to extend human life expectancy, are limited. The critical question is as follows: what are these pathogenic pathways, and how can they be effectively countered for therapeutic purposes?

Human cells contain >500 protein kinases and nearly 200 protein phosphatases acting on thousands of proteins including cell growth factors in health and disease; see [3,4]. At present, BDNF, NGF, and pro-NGF play a crucial role in the pathogenesis of a wide spectrum of neuronal and non-neuronal disorders, such as Alzheimer’s and other neurodegenerative disorders, including obesity and related CMD [3,4,6]. The decreased presence of resident and/or blood circulating BDNF and NGF was described in metabolic syndrome, human coronary atherosclerosis, and acute coronary syndromes [3,4,5,6,7,9,10], suggestive of (i) a key function played by BDNF and NGF in the pathogenetic processes and (ii) a potential therapeutic action of TrkB^BDNF^ and TrkA^NGF^ receptor agonists in CMD. Indeed, it is well known that BDNF acts in the leptin-mediated anorexigenic circuit to regulate the adipose-brain regulation of food intake; see [5,6]. Mice heterozygous for BDNF-targeted disruption and mice with a reduced expression of the TrkB^BDNF^ receptor show hyperphagia and obesity; see [4,5,6].

Notably, short-term myocardial ischemia produces a sympathetic cardiac innervation dysfunction associated with a rapid elevation in NGF release, while the NGF exogenous administration acts against such neuronal dysfunction, indicating that the endogenous production of NGF is inadequate for efficient neural protection [75]. Since reduced local and/or circulating levels of NGF and BDNF were found to be related to atherogenesis [3,4,5,6,7,8,9,10], the stimulation of TkrA^NGF^ and TrkB^BDNF^ receptors could create possible agonistic trackins with an anti-atherosclerotic effect.

Furthermore, recent studies show the therapeutic potential of NGF in the healing of corneal and cutaneous wounds [8,28,62,63,76,77], while TrkA^NGF^ receptor antagonists have been studied for new drugs for prostate, breast, and other malignant tumors, as well as for pain [20,29,30,78]. Stromal cells of the prostate and adipose stromal cells secrete NGF, which, in a paracrine way, can stimulate the carcinogenic proliferation of prostatic epithelial cells [79]. In support of such data, chemical substances that inhibit TrkA^NGF^ receptors are increasingly being investigated as potential anticancer drugs. For instance, TrkA^NGF^ receptor expression is positively associated with the invasion and malignancy of cancer cells in the prostate, and its antagonist Lestaurtinib (codename CEP-701) was included in some clinical trials focusing on prostate cancer [80]. This drug is the chemical substance indolocarbazole that specifically inhibits the TrkA^NGF^ receptor [19,80]. It should be noted that natural antibodies against NGF are also present in intravenous gammaglobulin (IVIg), which may inhibit the in vitro migration of prostate cancer cells; see [81]. Intriguingly, it was reported that tamoxifen, prescribed to breast cancer patients, may inhibit TrkA^NGF^ phosphorylation and, respectively, the NGF-elicited proliferation of epithelial cells from breast cancer [82]. Reflecting on the phenomenon of repurposed drugs (such as aspirin and colchicine), the findings about tamoxifen align with numerous other instances where an old drug has been found to have a new use. 

Another “danger” arises from data showing that NGF-induced increases in the sympathetic innervation of the myocardium are implicated in the pathobiology of sudden cardiac death [83]. We consider these latter results as suggestive of a probable participation of NGF and its TrkA receptor in the pathogenetic mechanisms of arrhythmogenic right-ventricular dysplasia [24]. This is a genetic type of cardiomyopathy, characterized histologically by the substitution of deteriorated cardiomyocytes with NGF/BDNF-produced adipocytes documented in our immunohistochemical study [24]. Despite this, the possibility of TrkA^NGF^ and TrkC^NT−3^ receptor antagonists possessing an anti-arrhythmogenic action remains to further be investigated.

Intriguingly, high-pressure treatment with sterile physiological saline isotonic solution into the nasal cavity of individuals with sensorineural hearing loss and tinnitus potentiates NGF levels (in the nasal fluid), leading to improved hearing [84]. “Paradoxically”, recent experimental results obtained with a TrkA^NGF^ receptor inhibitor, GW441756, suggest that one component of an optimal therapy for Alzheimer’s disease may be a TrkA^NGF^ antagonist [20].

## 4. Conclusions and Perspectives

In science, the Apollonian tends to develop established lines to perfection, while the Dionysian rather relies on intuition and is more likely to open new, unexpected alleys for research. The future of mankind depends on the progress of science, and the progress of science depends on the support it can find. Support mostly takes the form of grants, and the present methods of distributing grants unduly favor the Apollonian.

Albert Szent-Gyorgyi (1972), Nobel Prize winner 1937 in Physiology or Medicine

This translational review highlighted the possible druggability of NGF-TrkA^NGF^-TrkA^pro-NGF^, BDNF-TrkB^BDNF^, and NT3-TrkC^NT−3^ through agonistic or antagonistic trackins for therapy for different pathologies (Table 4). This may contribute to the theoretical hypothesis of an innovative therapeutic frame for further translational investigations dealing with trackins.

Let us remember that the plasma membrane contains microdomains termed lipid rafts (LRs, existing as caveolae) that are enriched in lipids, such as glycosphingolipids, gangliosides, and cholesterol [86]; LRs are scaffolds for many receptors. Much evidence indicates that the functions of LRs depend upon the interactions with the cytoskeletal microtubules (MT) and MT-associated motor proteins [87]. NGF enhances the interaction between TrkA and MT at lipid rafts controlling different cellular responses including axonal growth [87]. These data suggest the existence of an *intriguing quartet* consisting of NGF-Trk^NGF^-MT-LR. In the brain, pro-NGF is the only detectable form of NGF; thus, the dysregulation of pro-NGF and/or its TrkA^pro-NGF^ receptor in the brain could be implicated in age-related memory loss, including AD [87]. Further, the current data suggest that an increase in reactive oxygen species (ROS) and reactive nitrogen species (RNS) reduces the expression of the TrkA^pro-NGF^ receptor; additionally, the dysfunction of the MT motors kinesin and dynein may lead to disruptions to the TrkA^pro-NGF^ receptor’s downstream survival signaling [88]. Conventional thinking immediately proposes antioxidant treatments as beneficial in restoring pro-NGF signaling and reducing brain neurodegeneration and related deficits in cognitive function. Since ROS-RNS also interferes with the abovementioned *intriguing quartet* [88], we wonder whether the murburn concept of the biology of oxygen [89,90,91] could explain such an association between Trk, ROS, RNS, MT, and AD.

Existing limits to Trk-targeting drug development include several critical tasks. One main issue is achieving a high specificity for Trk receptors without distressing similar kinases, leading to off-target results and unsolicited side effects. Furthermore, the progress of resistance mechanisms in the disease through mutations or unusual signaling pathways, obscures the long-term efficiency of these drugs. There are pharmacokinetic obstacles too, such as limited bioavailability and impairments in drug delivery to target tissues, restricting their therapeutic potential. Addressing these limits is crucial for advancing Trk-targeting treatments and improving outcomes for people with Trk-driven disorders.

***In a nutshell***

The concept of trackins highlighted herein is a promising step forward, but not the whole journey—however, it promises a reward in future translational research. Since 2016, see [3,4,5,7,22], we have been “pondering what no one else has yet considered about what everyone observes”, thus introducing the term trackins [4] with respect to the bivalent nature of the druggability of TrkA^NGF^ and TrkB^BDNF^ receptors and, consequently, their stimulation or inhibition by trackins (pharmaceuticals, nutraceuticals, and/or biologicals), showing the relevance of this subject to therapies for the different diseases discussed in the present short review.

Doubtless, we remember René Descartes’ idea that “*de omnibus dubitare, vel dubitare de ipsa*” (from Latin—“everything must be doubted”).

## 5. Addendum

Human love of knowledge leads to the wish to “see inside” the body of organisms. Initially, this was achieved by Aristotle’s biology, the first in the history of science, which included five major processes:

1. A metabolic process, whereby animals take in matter, change its qualities, and distribute these to use to grow, live, and reproduce.
2. Temperature regulation, whereby animals maintain a steady state, which progressively fails in old age.
3. An information-processing model, whereby animals receive sensory information and use it to drive movements of the limbs.
4. The process of inheritance.
5. The processes of embryonic development and spontaneous generation

**Figure 3:**
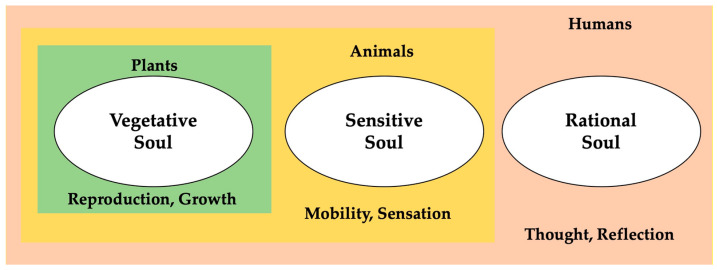
Structure of the soul of plants, animals, and humans. In this vision, humans are unique in having all three types of souls symbiotically. Here, it is reasonable to quote Socrates—“Man is a soul that serves his body”—as a first conceptual step to envisage the soul-and-body interaction [92].

These five processes formed what Aristotle (384–322 BC) called *the soul,* as illustrated in Figure 3:

## Figures and Tables

**Table 1:** Neurotrophin receptors and ligands. * Notably, the Trk receptor’s transactivation through the G protein-coupled receptor has lately arisen as an original perspective on neurotrophin functions [12].

Receptors *	Neurotrophins
p75^NTR^	NGF, BDNF, NT-3. NT-4/5
TrkA	NGF, pro-NGF
TrkB	BDNF, pro-BDNF, NT-4/5
TrkC	NT-3

**Table 2:** Multiple effects of NGF and BDNF. * Arrhythmogenic right-ventricular dysplasia (ARVD) is characterized by the accumulation and dysfunction of adipose tissue in the right ventricle of the heart, leading to ventricular arrhythmias and progressive right-ventricular failure, and often sudden cardiac death.

Physiotherapeutic	Pathogenic
Neurotrophic [13,14,15,16]	Oncotrophic (cancerogenic) [17,18,19,20]
Immunotrophic [21,22]	Nociceptogenic [23]
Меtabotrophic [5,6,22]	Arrhythmogenic [24] *
Psychotrophic [20,25,26,27,28,29,30]	Pruritus [31,32]
Cognitogenic [33,34,35,36,37,38,39,40,41,42]	Dry-eye disease [43]
Angiogenic [44,45,46,47,48,49,50,51,52,53]	
Sperm vitality, mobility, fertility [54]	
Skin, cornea, axon and bone wound/fracture healing [31,32,43,55,56,57,58,59,60,61,62,63,64,65,66,67,68,69,70]	

**Table 3:** Metabotrophic effects of NGF and BDNF [5,6,22,54].

NGF and BDNF are released by pancreatic beta cells and have an insulinotropic effect
NGF has homology with proinsulin
BDNF-deficient mice may develop metabolic syndrome-like abnormalities
NGF up-regulates the expression of PPAR-gamma
NGF and BDNF are trophic factors for pancreatic beta cells
BDNF improves cognition
NGF up-regulates the expression of LDL receptor-related proteins
NGF increases skin and corneal wound healing
NGF inhibits glucose-induced down-regulation of caveolin-1
NGF increases diabetic erectile dysfunction
NGF may rescue silent myocardial ischemia in diabetes mellitus
A healthy lifestyle potentiates brain and/or circulating BDNF and NGF
An atherogenic diet reduces brain BDNF
BDNF potentiates cognitive processes
BDNF-deficient mice may develop abnormalities similar to the metabolic syndrome

**Table 4:** Trackins and therapy for different diseases; see [3,4,6,23,27,28,29,30,31,32,57,58,59,62,63,64,78,79,85]. * T2/3DM, type 2/3 diabetes mellitus.

Agonists	Antagonists
TrkANGF, TrkApro-NGF, TrkBBDNF, TrkCNT−3	TrkANGF
**Cardiometabolic diseases**	**Cancers**
Atherosclerosis, hypertension	Prostate, Breast
Obesity, T2DM *, metabolic syndrome	Brain, Pancreas, Lung
Atrial fibrillation	
Diabetic erectile dysfunction	
**Cardiovascular diseases**	
Arrhythmogenic right ventricular dysplasia	
Sudden cardiac death	
**Neurometabolic diseases**	
Alzheimer’s disease (T3DM) *	**Pain**
Parkinson’s disease	**Pruritus**
Multiple sclerosis	
**Wounds**	
Skin, cornea, bone, axon	

## Data Availability

Data sharing is not applicable.

## References

[1] Levi-Montalcini R. (1987). The nerve growth factor 35 years later. Science.

[2] Cohen S., Levi-Montalcini R., Hamburger V. (1954). A Nerve Growth-Stimulating Factor Isolated from Sarcomas 37 and 180. Proc. Natl. Acad. Sci. USA.

[3] Yanev S., Aloe L., Fiore M., Chaldakov G.N. (2013). Neurotrophic and metabotrophic potential of nerve growth factor and brain-derived neurotrophic factor: Linking cardiometabolic and neuropsychiatric diseases. World J. Pharmacol..

[4] Yanev S., Fiore M., Hinev A., Ghenev P.I., Hristova M.G., Panayotov P., Tonchev A., Evtimov N., Aloe L., Chaldakov G.N. (2016). From antitubulins to trackins. Biomed. Rev..

[5] Chaldakov G., Fiore M., Tonchev A., Aloe L. (2008). Adipopharmacology, a Novel Drug Discovery Approach: A Metabotrophic Perspective. Lett. Drug Des. Discov..

[6] Frohlich J., Chaldakov G.N., Vinciguerra M. (2021). Cardio- and Neurometabolic Adipobiology: Consequences and Implications for Therapy. Int. J. Mol. Sci..

[7] Chaldakov G.N., Fiore M., Stankulov I.S., Manni L., Hristova M.G., Antonelli A., Ghenev P.I., Aloe L. (2004). Neurotrophin presence in human coronary atherosclerosis and metabolic syndrome: A role for NGF and BDNF in cardiovascular disease?. Prog. Brain Res..

[8] Aloe L., Tirassa P., Lambiase A. (2008). The topical application of nerve growth factor as a pharmacological tool for human corneal and skin ulcers. Pharmacol. Res..

[9] Chaldakov G.N., Stankulov I.S., Fiore M., Ghenev P.I., Aloe L. (2001). Nerve growth factor levels and mast cell distribution in human coronary atherosclerosis. Atherosclerosis.

[10] Manni L., Nikolova V., Vyagova D., Chaldakov G.N., Aloe L. (2005). Reduced plasma levels of NGF and BDNF in patients with acute coronary syndromes. Int. J. Cardiol..

[11] Gentry J.J., Barker P.A., Carter B.D. (2004). The p75 neurotrophin receptor: Multiple interactors and numerous functions. Prog. Brain Res..

[12] Jeanneteau F., Chao M.V. (2006). Promoting neurotrophic effects by GPCR ligands. Novartis Found Symp..

[13] Sahay A., Kale A., Joshi S. (2020). Role of neurotrophins in pregnancy and offspring brain development. Neuropeptides.

[14] Galvez-Contreras A.Y., Campos-Ordonez T., Lopez-Virgen V., Gomez-Plascencia J., Ramos-Zuniga R., Gonzalez-Perez O. (2016). Growth factors as clinical biomarkers of prognosis and diagnosis in psychiatric disorders. Cytokine Growth Factor Rev..

[15] Lamballe F., Klein R., Barbacid M. (1991). The trk family of oncogenes and neurotrophin receptors. Princess Takamatsu Symp..

[16] French S.J., Humby T., Horner C.H., Sofroniew M.V., Rattray M. (1999). Hippocampal neurotrophin and trk receptor mRNA levels are altered by local administration of nicotine, carbachol and pilocarpine. Mol. Brain Res..

[17] Sinkevicius K.W., Kriegel C., Bellaria K.J., Lee J., Lau A.N., Leeman K.T., Zhou P., Beede A.M., Fillmore C.M., Caswell D. (2014). Neurotrophin receptor TrkB promotes lung adenocarcinoma metastasis. Proc. Natl. Acad. Sci. USA.

[18] Aloe L., Rocco M.L., Balzamino B.O., Micera A. (2016). Nerve growth factor: Role in growth, differentiation and controlling cancer cell development. J. Exp. Clin. Cancer Res..

[19] Esteban-Villarrubia J., Soto-Castillo J.J., Pozas J., San Román-Gil M., Orejana-Martín I., Torres-Jiménez J., Carrato A., Alonso-Gordoa T., Molina-Cerrillo J. (2020). Tyrosine kinase receptors in oncology. Int. J. Mol. Sci..

[20] Zhang Q., Descamps O., Hart M.J., Poksay K.S., Spilman P., Kane D.J., Gorostiza O., John V., Bredesen D.E. (2014). Paradoxical effect of TrkA inhibition in alzheimer’s disease models. J. Alzheimer’s Dis..

[21] Ciafrè S., Ferraguti G., Tirassa P., Iannitelli A., Ralli M., Greco A., Chaldakov G.N., Rosso P., Fico E., Messina M.P. (2020). Nerve growth factor in the psychiatric brain. Riv. Psichiatr..

[22] Chaldakov G.N. (2011). The metabotrophic NGF and BDNF: An emerging concept. Arch. Ital. Biol..

[23] Hirose M., Kuroda Y., Murata E. (2016). NGF/TrkA Signaling as a Therapeutic Target for Pain. Pain Pract..

[24] Ghenev P., Kitanova M., Popov H., Evtimov N., Stoev S., Tonchev A., Chaldakov G. (2017). Neuroadipobiology of arrhythmogenic right ventricular dysplasia. An immunohistochemical study of neurotrophins. Adipobiology.

[25] Voronin M.V., Vakhitova Y.V., Seredenin S.B. (2020). Chaperone Sigma1R and antidepressant effect. Int. J. Mol. Sci..

[26] Alizadeh Pahlavani H. (2024). Possible role of exercise therapy on depression: Effector neurotransmitters as key players. Behav. Brain Res..

[27] Hochstrasser T., Ehrlich D., Sperner-Unterweger B., Humpel C. (2013). Antidepressants and anti-inflammatory drugs differentially reduce the release of NGF and BDNF from rat platelets. Pharmacopsychiatry.

[28] Angelucci F., Mathé A.A., Aloe L. (2004). Neurotrophic factors and CNS disorders: Findings in rodent models of depression and schizophrenia. Prog. Brain Res..

[29] Kozisek M.E., Middlemas D., Bylund D.B. (2008). Brain-derived neurotrophic factor and its receptor tropomyosin-related kinase B in the mechanism of action of antidepressant therapies. Pharmacol. Ther..

[30] Jiang C., Salton S.R. (2013). The role of neurotrophins in major depressive disorder. Transl. Neurosci..

[31] Raap U., Ständer S., Metz M. (2011). Pathophysiology of itch and new treatments. Curr. Opin Allergy Clin. Immunol..

[32] Raap U., Papakonstantinou E., Metz M., Lippert U., Schmelz M. (2016). Update on the cutaneous neurobiology of pruritus. Hautarzt.

[33] Zisiadis G.A., Alevyzaki A., Nicola E., Rodrigues C.F.D., Blomgren K., Osman A.M. (2023). Memantine increases the dendritic complexity of hippocampal young neurons in the juvenile brain after cranial irradiation. Front. Oncol..

[34] Minoretti P., Santiago Sáez A.S., García Martín Á.F., Riera M., Gómez Serrano M., Lahmar A., Emanuele E. (2023). Impact of Job Types on Plasma Neurotrophins Levels: A Preliminary Study in Airline Pilots, Construction Workers, and Fitness Instructors. Neuro Endocrinol. Lett..

[35] Khodabakhsh P., Asgari Taei A., Shafaroodi H., Pournajaf S., Dargahi L. (2024). Effect of Metformin on Epidermal Neural Crest Stem Cells and Their Potential Application in Ameliorating Paclitaxel-induced Neurotoxicity Phenotype. Stem Cell Rev. Rep..

[36] Chen W., Ren Q., Zhou J., Liu W. (2024). Mesenchymal Stem Cell-Induced Neuroprotection in Pediatric Neurological Diseases: Recent Update of Underlying Mechanisms and Clinical Utility. Appl. Biochem. Biotechnol..

[37] Moghadasi M., Akbari F., Najafi P. (2024). Interaction of aerobic exercise and crocin improves memory, learning and hypocampic tau and neurotrophins gene expression in rats treated with trimethytin as a model of Alzheimer’s disease. Mol. Biol. Rep..

[38] Shafiee A., Rafiei M.A., Jafarabady K., Eskandari A., Abhari F.S., Sattari MAAmini M.J., Bakhtiyari M. (2024). Effect of cannabis use on blood levels of brain-derived neurotrophic factor (BDNF) and nerve growth factor (NGF): A systematic review and meta-analysis. Brain Behav..

[39] Tu Y., Han D., Liu Y., Hong D., Chen R. (2024). Nicorandil attenuates cognitive impairment after traumatic brain injury via inhibiting oxidative stress and inflammation: Involvement of BDNF and NGF. Brain Behav..

[40] Liu J., Guan J., Xiong J., Wang F. (2024). Effects of Transcranial Magnetic Stimulation Combined with Sertraline on Cognitive Level, Inflammatory Response and Neurological Function in Depressive Disorder Patients with Non-suicidal Self-injury Behavior. Actas Esp. Psiquiatr..

[41] Crews F.T., Macht V., Vetreno R.P. (2024). Epigenetic regulation of microglia and neurons by proinflammatory signaling following adolescent intermittent ethanol (AIE) exposure and in human AUD. Adv. Drug Alcohol. Res..

[42] Sun L., Xiao K., Shen X.-Y., Wang S. (2024). Impact of transcranial electrical stimulation on serum neurotrophic factors and language function in patients with speech disorders. World J. Clin. Cases.

[43] Yu Z., Joy S., Mi T., Yazdanpanah G., Burgess K., de Paiva C.S. (2022). New, potent, small molecule agonists of tyrosine kinase receptors attenuate dry eye disease. Front. Med..

[44] Ricci A., Salvucci C., Castelli S., Carraturo A., de Vitis C., D’Ascanio M. (2022). Adenocarcinomas of the Lung and Neurotrophin System: A Review. Biomedicines.

[45] Sullivan I., Kc R., Singh G., Das V., Ma K., Li X., Mwale F., Votta-Velis G., Bruce B., Natarajan Anbazhagan A. (2022). Sensory Neuron-Specific Deletion of Tropomyosin Receptor Kinase A (TrkA) in Mice Abolishes Osteoarthritis (OA) Pain via NGF/TrkA Intervention of Peripheral Sensitization. Int. J. Mol. Sci..

[46] Qin S., Zhang Z., Zhao Y., Liu J., Qiu J., Gong Y., Fan W., Guo Y., Guo Y., Xu Z. (2022). The impact of acupuncture on neuroplasticity after ischemic stroke: A literature review and perspectives. Front. Cell Neurosci..

[47] Arutjunyan A.V., Kerkeshko G.O., Milyutina Y.P., Shcherbitskaia A.D., Zalozniaia I.V., Mikhel A.V., Inozemtseva D.B., Vasilev D.S., Kovalenko A.A., Kogan I.Y. (2023). Imbalance of Angiogenic and Growth Factors in Placenta in Maternal Hyperhomocysteinemia. Biochemistry.

[48] Lutfi Ismaeel G., Makki AlHassani O.J., Alazragi R.S., Hussein Ahmed A., Mohamed A.H., Yasir Jasim N., Hassan Shari F., Almashhadani H.A. (2023). Genetically engineered neural stem cells (NSCs) therapy for neurological diseases; state-of-the-art. Biotechnol. Prog..

[49] Redigolo L., Sanfilippo V., La Mendola D., Forte G., Satriano C. (2023). Bioinspired Nanoplatforms Based on Graphene Oxide and Neurotrophin-Mimicking Peptides. Membranes.

[50] Jashire Nezhad N., Safari A., Namavar M.R., Nami M., Karimi-Haghighi S., Pandamooz S., Dianatpour M., Azarpira N., Khodabandeh Z., Zare S. (2023). Short-term beneficial effects of human dental pulp stem cells and their secretome in a rat model of mild ischemic stroke. J. Stroke Cerebrovasc. Dis..

[51] Pahlavani H.A. (2023). Exercise therapy to prevent and treat Alzheimer’s disease. Front. Aging Neurosci..

[52] Wan T., Zhang F.S., Qin M.Y., Jiang H.R., Zhang M., Qu Y., Wang Y.L., Zhang P.X. (2024). Growth factors: Bioactive macromolecular drugs for peripheral nerve injury treatment—Molecular mechanisms and delivery platforms. Biomed. Pharmacother..

[53] Giri S.S., Tripathi A.S., Erkekoğlu P., Zaki M.E.A. (2024). Molecular pathway of pancreatic cancer-associated neuropathic pain. J. Biochem. Mol. Toxicol..

[54] Stabile A.M., Pistilli A., Moretti E., Bartolini D., Ruggirello M., Rende M., Castellini C., Mattioli S., Ponchia R., Tripodi S.A. (2023). A Possible Role for Nerve Growth Factor and Its Receptors in Human Sperm Pathology. Biomedicines.

[55] Rosso P., Fico E., Mesentier-Louro L.A., Triaca V., Lambiase A., Rama P., Tirassa P. (2021). NGF eye administration recovers the trkb and glutamate/GABA marker deficit in the adult visual cortex following optic nerve crush. Int. J. Mol. Sci..

[56] D’Souza S., Vaidya T., Nair A.P., Shetty R., Kumar N.R., Bisht A., Panigrahi T., J T.S., Khamar P., Dickman M.M. (2022). Altered Ocular Surface Health Status and Tear Film Immune Profile Due to Prolonged Daily Mask Wear in Health Care Workers. Biomedicines.

[57] Shu J., He X., Li H., Liu X., Qiu X., Zhou T., Wang P., Huang X. (2018). The beneficial effect of human amnion mesenchymal cells in inhibition of inflammation and induction of neuronal repair in EAE mice. J. Immunol. Res..

[58] Sun S., Diggins N.H., Gunderson Z.J., Fehrenbacher J.C., White F.A., Kacena M.A. (2020). No pain, no gain? The effects of pain-promoting neuropeptides and neurotrophins on fracture healing. Bone.

[59] Valente S., Curti N., Giampieri E., Randi V., Donadei C., Buzzi M., Versura P. (2022). Impact of blood source and component manufacturing on neurotrophin content and in vitro cell wound healing. Blood Transfus..

[60] Sipione R., Liaudet N., Rousset F., Landis B.N., Hsieh J.W., Senn P. (2023). Axonal Regrowth of Olfactory Sensory Neurons In Vitro. Int. J. Mol. Sci..

[61] Zhang Y., Zhao X., Ge D., Huang Y., Yao Q. (2024). The impact and mechanism of nerve injury on bone metabolism. Biochem. Biophys. Res. Commun..

[62] Lambiase A., Rama P., Bonini S., Caprioglio G., Aloe L. (1998). Topical Treatment with Nerve Growth Factor for Corneal Neurotrophic Ulcers. N. Engl. J. Med..

[63] Aloe L. (2004). Nerve growth factor, human skin ulcers and vascularization. Our experience. Prog. Brain Res..

[64] Raap U., Kapp A. (2010). Neurotrophins in healthy and diseased skin. G. Ital. Dermatol. Venereol. Organo Uff. Soc. Ital. Dermatol. Sifilogr..

[65] Scuri M., Samsell L., Piedimonte G. (2010). The role of neurotrophins in inflammation and allergy. Inflamm. Allergy Drug Targets.

[66] D’Amico F., Lugarà C., Luppino G., Giuffrida C., Giorgianni Y., Patanè E.M., Manti S., Gambadauro A., La Rocca M., Abbate T. (2024). The Influence of Neurotrophins on the Brain-Lung Axis: Conception, Pregnancy, and Neonatal Period. Curr. Issues Mol. Biol..

[67] Pham T.L., He J., Kakazu A.H., Jun B., Bazan N.G., Bazan H.E.P. (2017). Defining a mechanistic link between pigment epithelium–derived factor, docosahexaenoic acid, and corneal nerve regeneration. J. Biol. Chem..

[68] Ghosh T., Maity N., Sur V.P., Konar A., Hazra S. (2020). Mitigating fibrosis-An impediment to corneal re-innervation following lamellar flap surgery. Exp. Eye Res..

[69] Micera A., Jirsova K., Esposito G., Balzamino B.O., Zazzo ADi Bonini S. (2020). Mast cells populate the corneoscleral limbus: New insights for our understanding of limbal microenvironment. Investig. Ophthalmol. Vis. Sci..

[70] Moramarco A., Sacchetti M., Franzone F., Segatto M., Cecchetti D., Miraglia E., Roberti V., Iacovino C., Giustini S. (2020). Ocular surface involvement in patients with neurofibromatosis type 1 syndrome. Graefe’s Arch. Clin. Exp. Ophthalmol..

[71] Owens J. (2007). Determining druggability. Nat. Rev. Drug Discov..

[72] Dixon S.J., Stockwell B.R. (2009). Identifying druggable disease-modifying gene products. Curr. Opin. Chem. Biol..

[73] Carito V., Ceccanti M., Cestari V., Natella F., Bello C., Coccurello R., Mancinelli R., Fiore M. (2017). Olive polyphenol effects in a mouse model of chronic ethanol addiction. Nutrition.

[74] Fiore M., Messina M.P., Petrella C., D’Angelo A., Greco A., Ralli M., Ferraguti G., Tarani L., Vitali M., Ceccanti M. (2020). Antioxidant properties of plant polyphenols in the counteraction of alcohol-abuse induced damage: Impact on the Mediterranean diet. J. Funct. Foods.

[75] Abe T., Morgan D.A., Gutterman D.D. (1997). Protective role of nerve growth factor against postischemic dysfunction of sympathetic coronary innervation. Circulation.

[76] Aloe L., Rocco M.L., Bianchi P., Manni L. (2012). Nerve growth factor: From the early discoveries to the potential clinical use. J. Transl. Med..

[77] Chiaretti A., Piastra M., Caresta E., Nanni L., Aloe L. (2002). Improving ischaemic skin revascularisation by nerve growth factor in a child with crush syndrome. Arch. Dis. Child.

[78] Watanabe T., Inoue M., Sasaki K., Araki M., Uehara S., Monden K., Saika T., Nasu Y., Kumon H., Chancellor M.B. (2011). Nerve growth factor level in the prostatic fluid of patients with chronic prostatitis/chronic pelvic pain syndrome is correlated with symptom severity and response to treatment. BJU Int..

[79] Warrington R.J., Lewis K.E. (2011). Natural antibodies against nerve growth factor inhibit in vitro prostate cancer cell metastasis. Cancer Immunol. Immunother..

[80] Festuccia C., Muzi P., Gravina G.L., Millimaggi D., Speca S., Dolo V., Ricevuto E., Vicentini C., Bologna M. (2007). Tyrosine kinase inhibitor CEP-701 blocks the NTRK1/NGF receptor and limits the invasive capability of prostate cancer cells in vitro. Int. J. Oncol..

[81] Thiele C.J., Li Z., McKee A.E. (2009). On Trk—The TrkB signal transduction pathway is an increasingly important target in cancer biology. Clin. Cancer Res..

[82] Chiarenza A., Lazarovici P., Lempereur L., Cantarella G., Bianchi A., Bernardini R. (2001). Tamoxifen inhibits nerve growth factor-induced proliferation of the human breast cancerous cell line MCF-7. Cancer Res..

[83] Chen P.S., Chen L.S., Cao J.M., Sharifi B., Karagueuzian H.S., Fishbein M.C. (2001). Sympathetic nerve sprouting, electrical remodeling and the mechanisms of sudden cardiac death. Cardiovasc. Res..

[84] Salvinelli F., Frari V., Rocco M.L., Rosso P., Aloe L. (2015). Enhanced presence of NGF and mast cells number in nasal cavity after autologous stimulation: Relation with sensorineural hearing deficit. Eur. Rev. Med. Pharmacol. Sci..

[85] Povarnina P.Y., Vorontsova O.N., Gudasheva T.A., Ostrovskaya R.U., Seredenin S.B. (2013). Original nerve growth factor mimetic dipeptide GK-2 restores impaired cognitive functions in rat models of Alzheimer’s disease. Acta Naturae.

[86] Head B.P., Patel H.H., Insel P.A. (2014). Interaction of membrane/lipid rafts with the cytoskeleton: Impact on signaling and function: Membrane/lipid rafts, mediators of cytoskeletal arrangement and cell signaling. Biochim. Biophys. Acta Biomembr..

[87] Pryor S., McCaffrey G., Young L.R., Grimes M.L. (2012). NGF causes TrKA to specifically attract microtubules to lipid rafts. PLoS ONE.

[88] Kropf E., Fahnestock M. (2021). Effects of reactive oxygen and nitrogen species on trka expression and signalling: Implications for prongf in aging and alzheimer’s disease. Cells.

[89] Manoj K.M. (2023). Murburn concept: Murzyme roles of redox proteins in xenobiotic metabolism and ATP-synthesis. Biomed. Rev..

[90] Manoj K.M., Jaeken L. (2023). Synthesis of theories on cellular powering, coherence, homeostasis and electro-mechanics: Murburn concept and evolutionary perspectives. J. Cell Physiol..

[91] Francati S., Fiore M., Ferraguti G. (2023). The janus face of oxidative stress in health and disease: The cause or the cure?. Biomed. Rev..

[92] Lurz J., Ladwig K.H. (2022). Mind and body interventions in cardiology: The importance of the brain–heart connection. Herz.

